# Structural modeling and biochemical characterization of MSMEG_0748, a MoxR ATPase from *Mycobacterium smegmatis*

**DOI:** 10.1016/j.csbj.2025.11.010

**Published:** 2025-11-07

**Authors:** Ju Eun Lee, So Yeon Lee, Hyo Guk Kim, Minkyung Cho, Hyun-A. Seong, Sang-Min Jang, Hyo Je Cho

**Affiliations:** a Department of Biological Sciences and Biotechnology, Chungbuk National University, Cheongju 28644, Republic of Korea; b Department of Biochemistry, Chungbuk National University, Cheongju 28644, Republic of Korea

**Keywords:** *Mycobacterium smegmatis*, MoxR ATPase, AlphaFold structure prediction, ATPase assay, CO-DH maturation

## Abstract

*Mycobacterium tuberculosis* persists in a latent state within host macrophages by adapting to hostile conditions such as hypoxia and oxidative stress. Central to this adaptation, the carbon monoxide dehydrogenase (CO-DH) gene cluster supports intracellular survival by metabolizing carbon monoxide and nitric oxide. Among the uncharacterized genes within this cluster*, msmeg_0748*, annotated as a MoxR-type AAA+ ATPase in *Mycobacterium smegmatis* MC^2^ 155, is hypothesized to act as a chaperone in CO-DH maturation. To characterize this protein, we employed AlphaFold3 structure prediction, validated by sequence alignment, SEC-MALS, biochemical assays, and biophysical analyses. MSMEG_0748 was confirmed to form a characteristic hexameric structure in solution. The structural models displayed conserved features including Walker A and B motifs and the MoxR-ATPase specific Helix 2 Insert (H2I). Furthermore, ATP/NADH- enzyme coupled assays confirmed its intrinsic ATP hydrolysis activity, which was dependent on the conserved catalytic glutamate (E146) in the Walker B motif. Co-immunoprecipitation experiments subsequently demonstrated a direct interaction between MSMEG_0748 and the adjacent VWA domain-containing protein, MSMEG_0751. These results provide comprehensive structural and functional insights into MSMEG_0748 and suggest the MSMEG_0748–MSMEG_0751 complex functions as the MoxR–VWA chaperone system essential for metalloenzyme maturation in mycobacteria.

## Highlights

• MSMEG_0748 is confirmed as a functional hexameric MoxR-type ATPase.
• AlphaFold 3-predicted structure validated the high-confidence holo-hexamer.
• Catalytic residue E146 is critical for its intrinsic ATP hydrolysis activity.
• MSMEG_0748–MSMEG_0751 complex is implicated in CO-DH maturation.

## Graphical Abstract

**Figure:**
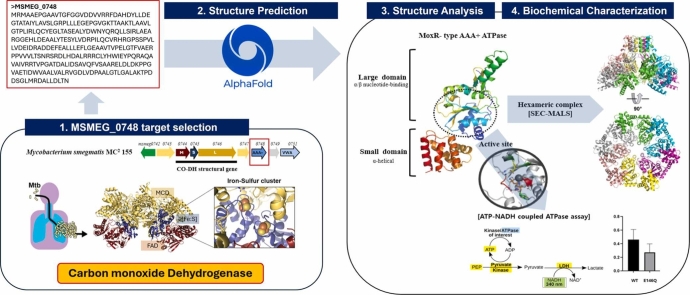

## 1 Introduction

AAA+ ATPases are a diverse superfamily of P-loop NTPases that play key roles in various cellular processes [1], including protein unfolding, disaggregation, complex remodeling, and the assembly of macromolecular complexes [2]. These enzymes usually function as hexameric ring structures, catalyzing ATP hydrolysis through conserved Walker A and Walker B motifs, which are essential for ATP binding and hydrolysis, respectively [3], [4]. Notably, a highly conserved glutamate residue within the Walker B motif is vital for ATP hydrolysis, facilitating a nucleophilic attack on the γ-phosphate of ATP [5], [6]. Structurally, AAA+ ATPases share a conserved N-terminal α/β nucleotide-binding domain and a C-terminal α-helical domain [7]. The MoxR subfamily is distinguished by the presence of both a pre-sensor 1 β-hairpin and a helix-2 insert, which contribute to substrate recognition and regulation during ATP-dependent remodeling [8]. Functionally, MoxR ATPases exhibit chaperone-like activities, assisting in the structural and functional maturation of target proteins [9]. They are frequently encoded in operons alongside von Willebrand factor type A (VWA) domain-containing proteins [10]. The MoxR–VWA complex is thought to mediate conformational remodeling or facilitate cofactor incorporation, which are critical steps in enzymatic maturation processes [11], [12], [13].

**Fig. 1:**
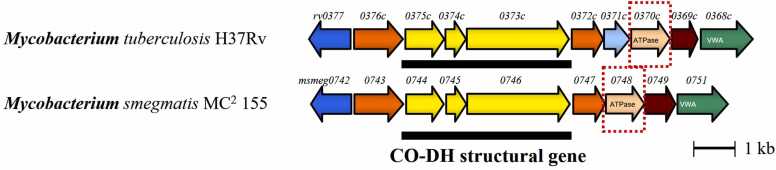
**Organization of CO-DH structural gene and conserved neighbour genes.** CO-DH gene cluster in *Mycobacterium tuberculosis* H37Rv (upper) and *M. smegmatis* (down). The genes for CO-DH genes are underlined. The genes for Rv0371c and MSMEG_0748 are boxed.

In *Mycobacterium tuberculosis*, maintaining the ability to persist in a latent state within host macrophages is critical for its survival and pathogenicity [14], [15]. To adapt to intracellular stress conditions, the bacterium relies on various metabolic pathways, one of which involves carbon monoxide dehydrogenase (CO-DH) [16]. This metalloprotein complex is critical for metabolizing carbon monoxide as an energy source during latency [17], [18]. CO-DH requires several cofactors for proper functioning, including molybdenum-containing molybdopterin cytosine dinucleotide (MCD), flavin adenine dinucleotide (FAD), and iron-sulfur protein with two [2Fe-2S] clusters [19]. Interestingly, genes encoding putative MoxR ATPase and von Willebrand factor type A (VWA) domain-containing proteins are consistently found immediately downstream of the CO-DH gene cluster, suggesting a possible cooperative role in the assembly and activation of the CO-DH complex (Fig. 1). Understanding the specific functions of these proteins could reveal new insights into their contributions to the stabilization or cofactor incorporation, which is crucial for the pathogen survival strategy. However, their precise roles remain poorly understood, requiring further investigation into their mechanisms and biological significance.

In this study, we initially attempted to characterize Rv0370c, a candidate MoxR-type AAA+ ATPase from *M. tuberculosis*. However, poor protein expression levels and solubility issues hampered the purification and functional analysis. Although we obtained initial crystals of *M. tuberculosis* Rv0370c, the poor diffraction quality prevented high-resolution structure determination, indicating that alternative strategies were required. To overcome this, we identified and successfully purified MSMEG_0748, a homolog of Rv0370c from *Mycobacterium smegmatis*. Nonetheless, structural analysis remained challenging due to difficulties in obtaining suitable, stable protein samples for both X-ray crystallography and Bio-SAXS experiments. Therefore, we employed the AlphaFold protein structure prediction platform, known for its high accuracy in similar systems, to generate a high-confidence structural model. This predicted structural model was validated and further supported by biochemical assays designed to access its functional properties. Based on structural features, sequence conservation, and genomic context, we propose that MSMEG_0748 functions as a MoxR ATPase involved in the maturation of the CO-DH complex. These findings provide a foundation for future investigations into the molecular interactions between MoxR and VWA proteins and their roles in enzyme assembly and cofactor incorporation, providing new avenues for understanding mycobacterial survival mechanisms and potential therapeutic targets.

## 2 Materials and methods

### 2.1 Cloning, expression, and purification of MSMEG_0748

The gene encoding MSMEG_0748 (UniProt ID: A0QQG6) was amplified by PCR from the genomic DNA of *Mycobacterium smegmatis* mc^2^ 155. The amplified fragment was subcloned into the pProEX-HTa vector containing an N-terminal His-tag followed by a recombinant TEV (rTEV) protease cleavage site. To generate the Walker B (E146Q) mutant, overlap PCR was performed using the wild-type MSMEG_0748 plasmid as a template. The resulting recombinant plasmids were transformed into *Escherichia coli* BL21(DE3) cells, which were cultured at 37°C in 4 L of LB medium supplemented with 50 μg/mL ampicillin. Protein expression was induced at an OD_600_ of approximately 0.6 by adding IPTG to a final concentration of 0.2 mM, and cells were incubated for an additional 20 h at 18°C after induction. Cells were harvested by centrifugation at 4000 × g for 20 min at 4°C. The cell pellet was resuspended in ice-cold buffer A (50 mM Tris-HCl, pH 7.5, 150 mM NaCl) and lysed by sonication. Cellular debris was removed by centrifugation at 13,000 × g for 40 min, and the clarified supernatant was loaded onto a Ni-NTA agarose column. After washing with buffer A containing 30 mM imidazole, the protein was eluted using buffer A supplemented with 200 mM imidazole. The eluted protein was treated with TEV protease during dialysis at 16°C for 16 h to remove the N-terminal His-tag. TEV protease and cleaved His-tag fragments were subsequently removed by reapplying the sample to the Ni-NTA column. Final purification was carried out using size-exclusion chromatography on a HiLoad™ 16/600 Superdex 200 pg column equilibrated with 20 mM Tris-HCl. pH 7.5 containing 150 mM NaCl.

### 2.2 SEC-MALS analysis

Size-exclusion chromatography with multi-angle light scattering (SEC-MALS) was performed using a Superdex 200 Increase 10/300 GL column at the Korea Basic Science Institute (KBSI). Purified MSMEG_0748 protein at 5 mg/mL was injected into the column, which was pre-equilibrated with buffer consisting of 20 mM Tris-HCl, pH 7.5, 300 mM NaCl, and 0.5 mM TCEP. Light scattering (LS), UV absorbance at 280 nm, and refractive index (RI) were simultaneously monitored. The molecular weight and oligomeric state were determined using a DAWN Heleos II detector.

### 2.3 MSMEG_0748 structure prediction using AlphaFold3

The promoter, dimer, trimer, and hexameric structures of MSMEG_0748, including the protomer structure in complex with ATP and Mg²⁺ and its hexameric assembly, were predicted using the AlphaFold3 server (https://alphafoldserver.com/) [20]. The generated structures were evaluated using pLDDT and the PAE scores [21]. The PAE graphs were visualized by the PAE viewer website, and the structures were visualized and analyzed using PyMol software.

### 2.4 ATPase activity assay (ATP/NADH coupled assay)

ATPase activity of wild-type MSMEG_0748 and its E146Q mutant was determined using an ATP/NADH coupled assay. Reactions were performed in a buffer containing 50 mM Tris-HCl, pH 8.0, 150 mM KCl, 2 mM MgCl2, 2 mM DTT, 9 mM phosphoenolpyruvate (PEP), 150 μM NADH, 60 U/mL pyruvate kinase, and 32 U/mL lactate dehydrogenase. Proteins were added at a final concentration of 200 nM. Reactions were initiated by adding ATP, AMP-PNP, and ADP to a final concentration of 100 μM. AMP-PNP, a non-hydrolyzable ATP analog, was included to assess binding effects independent of hydrolysis, while ADP, the reaction product, served to evaluate potential feedback inhibition or regulatory effects. The reactions were pre-incubated at 30°C for 10 min, followed by continuous monitoring of NADH oxidation at 340 nm for an additional 40 min using a SpectraMax iD3 microplate reader. Kinetic parameters were calculated by fitting data to the Michaelis-Menten equation using GraphPad Prism 10 software.

### 2.5 Transient transfection and Co-immunoprecipitation (Co-IP)

HEK293T cells were seeded in 6-well plates at a density of 3 × 10^5 cells/well and incubated for 24 h at 37°C in a CO2 incubator. Transfection was performed using the calcium phosphate method. Briefly, plasmid DNA was mixed with sterile distilled water and 2 M CaCl_2_, then added dropwise to 2x HBS buffer with gentle bubbling to facilitate precipitate formation. After 15 min of incubation at room temperature, the mixture was added dropwise to the cells. The medium was replaced after 6–8 h, and cells were harvested 48 h post-transfection for subsequent analysis. For Co-IP assays, transfected HEK293T cells were washed with cold PBS, lysed in NP-40 buffer for 30 min at 4°C, and centrifuged to remove insoluble debris. The cleared lysates were incubated with either anti-HA (Santa Cruz Biotechnology) or anti-FLAG antibody (Sigma-Aldrich) for 3 h at 4°C. Protein A–Sepharose beads (Cytiva) were then added, and the complexes were incubated for 1 h at 4°C. The immune complexes were washed several times with lysis buffer, then subjected to immunoblotting using ECL detection (ATTO Corporation).

## 3 Results and discussion

### 3.1 MSMEG_0748 exhibits conserved structural features of MoxR AAA+ ATPases

**Fig. 2:**
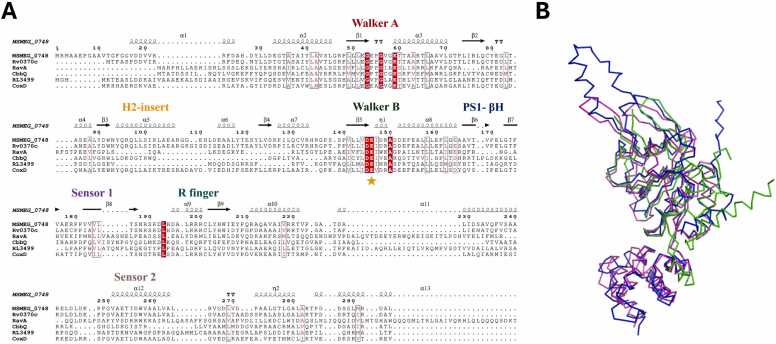
Sequence and structural alignment of MSMEG_0748 with representative MoxR ATPase. (A) Multiple sequence alignment highlighting conserved regions. Secondary structural elements, predicted for MSMEG_0748, are indicated above the sequences. Residues identical across all aligned sequences are shown with white text on a red background, while regions with > 70 % similarity (as determined by the Risler scoring matrix) are boxed in red le. conserved motif is labeled in text, and the yellow stars denote the main catalytic residues. The alignment was generated using ESPript (http://espript.ibcp.fr/ESPript/ESPript/). (B) Structural superposition of MSMEG_0748 (blue), *E. coli* RavA (green), and *A. ferrooxidans* CbbQ (magenta) shown in Cα representation.

To identify conserved structural features, we conducted a multiple sequence alignment of MSMEG_0748 with representative MoxR AAA+ ATPases. These included RavA from *Escherichia coli* (UniProt ID: P31473), CbbQ from *Acidithiobacillus ferrooxidans* (UniProt ID: B7J5E4), RL3499 from *Rhizobium johnstonii* (UniProt ID: Q1MDJ0), and CoxD from *Oligotropha carboxidovorans* (UniProt ID: Q51326). The alignment demonstrated that MSMEG_0748 shares the characteristic structural features of AAA+ ATPases. Notably, the ATP-binding and hydrolysis motifs, Walker A and Walker B, were highly conserved, consistent with other members of the MoxR-type AAA+ ATPase superfamily. Furthermore, the Helix 2 Insert (H2I), a structural feature characteristic of the MoxR subfamily, was observed in MSMEG_0748. Predicted to consist of two β-strands, this feature was also conserved, providing additional support for classifying MSMEG_0748 as a typical MoxR-type AAA+ ATPase (Fig. 2A). To further confirm structural homology, we performed structural superposition of the predicted AAA+ module of MSMEG_0748 onto the AAA+ modules of RavA (PDB ID: 6SZB) and CbbQ (PDB ID: 6L1Q), both of which have resolved structures. This superposition revealed that the AAA+ modules of these MoxR ATPases possess highly similar overall three-dimensional folds (Fig. 2B). Quantitatively, the root mean square deviation (RMSD) calculated over the Cα atoms of the aligned domains was 3.226 Å against RavA and 2.330 Å against CbbQ, strongly reinforcing the structural the classification of MSMEG_0748 as a structural homolog within the MoxR AAA+ ATPase family.

### 3.2 MSMEG_0748 forms a hexameric structure in solution

**Fig. 3:**
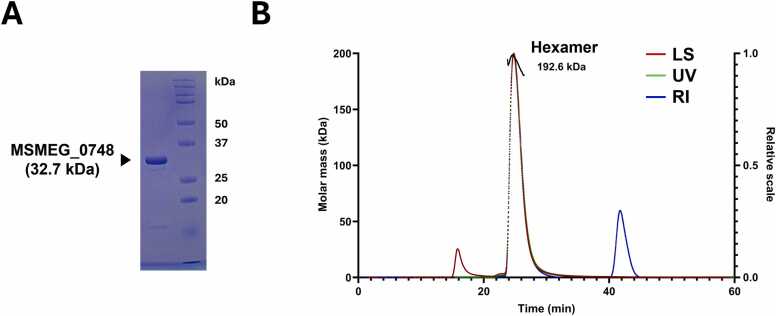
**Purification and SEC-MALS analysis of MSMEG_0748.** (A) The purified protein is indicated by the black arrow. 12 μg of protein was loaded on 12 % SDS-PAGE gel. B) Representative SEC-MALS analysis of purified MSMEG_0748, performed on a Superdex 200 Increase 10/300 GL column at a flow rate of 0.5 mL/min. The approximate Void Volume for this column is about 7.8 mL (about 15.6 min). The elution profile shows a single, symmetrical peak eluting between 23.8 and 26.4 min.

Recombinant MSMEG_0748 was successfully overexpressed in *E. coli* using the pProEX-HTa vector construct, which produces a fusion protein with a predicted molecular weight of approximately 35.74 kDa. The protein was purified using Ni-NTA affinity chromatography followed by size-exclusion chromatography. The purified protein was concentrated to 12 mg/mL, as measured by Bradford assay, and showed a high purity of > 95 % on SDS-PAGE analysis. A single protein band was observed between the 25 kDa and 37 kDa molecular weight standards, closely matching the expected size of the full-length MSMEG_0748 protein (∼32.7 kDa) after removal of the N-terminal His tag by rTEV protease (Fig. 3A). Since most AAA+ ATPases function as hexameric rings, we evaluated the oligomeric state of MSMEG_0748 using size-exclusion chromatography coupled with multi-angle light scattering (SEC-MALS). The purified protein eluted as a single peak, with an estimated molecular weight of approximately 192.6 kDa and a polydispersity index (M_w_/M_n_) of 1.001, indicating a highly homogeneous species in solution. This estimated mass is consistent with a hexameric assembly, considering its predicted monomeric mass of 32.7 kDa (corresponding to the tag-cleaved form) (Fig. 3B). These results support the formation of a hexamer in solution, which is characteristic of the quaternary structure of AAA+ ATPases. Although we successfully obtained crystals of MSMEG_0748, their diffraction quality was insufficient for structural elucidation. This likely reflects the intrinsic flexibility or dynamic behavior within the hexameric complex, which may interfere with optimal crystal packing.

### 3.3 AlphaFold predicts the 3D structure of MSMEG_0748

**Fig. 4:**
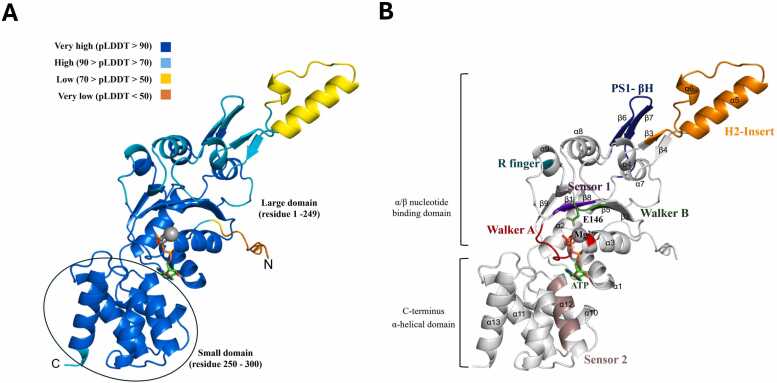
**Structure prediction of MSMEG_0748.** (A) AlphaFold-predicted model of MSMEG_0748 (https://alphafold.ebi.ac.uk/entry/A0QQG6). The pLDDT score provides a per-residue metric for assessing the confidence of the model predictions. (B) Structural features characteristic of the MoxR subfamily were observed in the predicted structure of MSMEG_0748, with features indicated in colors that are consistent with those shown in the sequence alignment in Fig. 2A.

To overcome the limitations of crystallographic analysis, we employed the AlphaFold server to predict the 3D structure of MSMEG_0748. The predicted monomer structure exhibited high overall confidence, with most regions displaying very high pLDDT scores, indicating reliable modeling. The overall fold corresponds to a typical AAA+ ATPase, comprising a large α/β nucleotide-binding domain and a C-terminal α-helical domain (Fig. 4A). Sequence motifs identified in the alignment were clearly mapped onto the structure. The Walker A motif, critical for ATP binding, is shown in red, while the Walker B motif, which contributes to ATP hydrolysis, is depicted in dark green. Both motifs are positioned in close proximity to the modelled ATP molecule (green) and a coordinating Mg²⁺ ion (gray sphere), consistent with their catalytic roles. A notable structural feature of the MoxR subfamily, the Helix 2 Insert (H2I) (orange), along with other characteristic elements such as PS1- βH (deep blue), Sensor 1 (purple), and Sensor 2 (raspberry), were also identified in the predicted model (Fig. 4B).

To provide quantitative validation for the hexameric state suggested by SEC-MALS and address the potential bias in oligomer prediction, we performed systematic AlphaFold3 predictions for MSMEG_0748 in various oligomeric states (monomer, dimer, trimer, and hexamer) and ligand conditions, evaluating the results using the interface Predicted Template Modeling (ipTM) and pTM scores. In the apo (ligand-free) state, the confidence for the hexameric assembly was moderate (ipTM = 0.57, pTM = 0.62), consistent with the inherent high flexibility of AAA+ ATPases in the absence of nucleotide. However, when the prediction was performed with bound ATP and Mg²⁺ (the holo state), the assembly confidence significantly improved: the ipTM score for the hexamer increased sharply to 0.74 (pTM = 0.77). This quantitative metric demonstrates that the hexameric interface is structurally stabilized and highly reliable only when the protein is in its functionally relevant, nucleotide-bound state, strongly supporting our biochemical findings. Furthermore, the core monomeric fold remained highly reliable across all conditions (pTM = 0.89 for Apo monomer, pTM = 0.85 for Holo monomer), indicating that the ligand primarily mediates conformational stability at the inter-subunit interfaces for stable hexamer formation rather than inducing major global changes in the protomer (Table 1).

**Table 1:** AlphaFold3 Prediction Metrics (ipTM and pTM) for the Oligomeric States of MSMEG_0748 and Ligand Effects.

Oligomeric State	Ligand Status	ipTM score(interface quality)	pTM score(overall quality)
Monomer	Apo	N/A	0.89
Dimer	Apo	0.74	0.80
Trimer	Apo	0.71	0.77
Hexamer	Apo	0.57	0.62
Monomer	Holo (ATP / Mg^2+^)	N/A	0.85
Hexamer	Holo (ATP / Mg^2+^)	0.74	0.77

To further benchmark the predicted hexamer against experimentally determined structures, we superimposed the AlphaFold-predicted holo-hexamer onto two known crystal structures of MoxR AAA+ ATPases: *E. coli* RavA (PDB ID: 6SZB) and *A. ferrooxidans* CbbQ (PDB ID: 6L1Q). The structural superposition demonstrated high conservation of the overall hexameric architecture. Specifically, the MSMEG_0748 hexamer exhibited an RMSD of 3.87 Å (168 Cα atoms) relative to the AAA+ ATPase domain (residues 3–211) of the RavA hexamer and 4.25 Å (248Cα atoms) relative to the CbbQ hexamer, as determined by CEalign. While lower RMSD values were observed upon comparison of the MSMEG_0748 monomeric structure with RavA and CbbQ monomers, these RMSD values for the hexameric assemblies provide strong quantitative validation for the suitability of our predicted MSMEG_0748 hexameric structure and highlight the accuracy with which it captures the conserved hexameric topology characteristic of the MoxR subfamily.

**Fig. 5:**
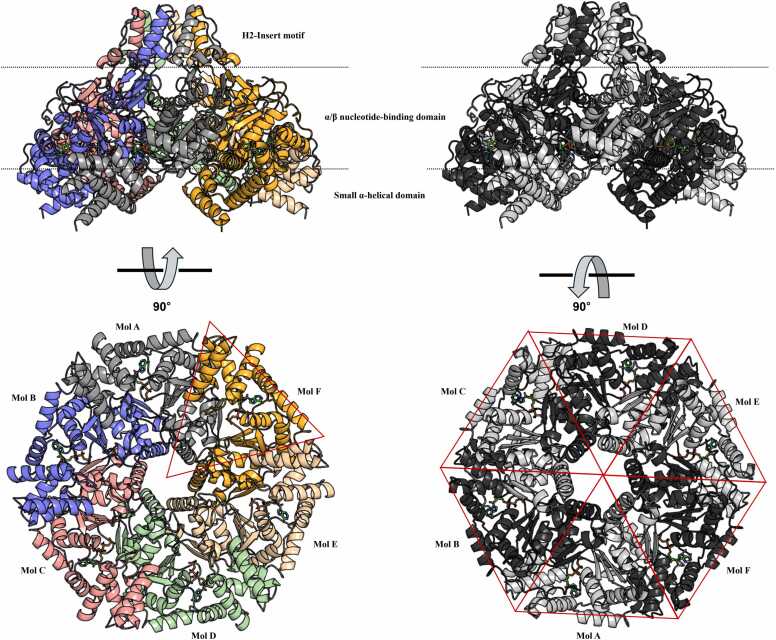
**Model of the MSMEG_0748 hexamer**. Side view of the hexamer highlighting the domain architecture of individual subunits: α/β nucleotide-binding domain, small α-helical domain, and H2-insert motif. Dashed lines indicate domain boundaries. Bound ATP molecules are shown as sticks. The same view in grayscale highlights the overall fold. The top view rotated by 90 °, with subunits (Mol A–Mol F) colored differently to illustrate six-fold symmetry; the grayscale top view, rotated by −90 °, shows the hexameric arrangement outlined in red triangle dashed line to depict hexagonal packing.

The hexameric assembly of MSMEG_0748 was further elucidated through symmetry modeling, revealing an arrangement where the H2I regions from adjacent subunits are positioned around the central pore. The hexameric ring displayed the expected architecture, with alternating large and small domains, consistent with previously solved MoxR-type structures. The symmetric arrangement of ATP-binding sites and the central cavity suggest coordinated conformational changes during catalysis, aligning with the behavior observed in other oligomeric, nucleotide-dependent enzymes. Furthermore, the crystal structure of *E. coli* RavA shows monomers packed in a left-handed helix, with ATP binding site at the interface between adjacent monomers within the helix [22]. Several other MoxR-type AAA+ ATPases crystallize as apparent monomers in space group *P65*, forming continuous helices due to crystal packing that resembles hexamers when viewed along the helical screw axis [23]. This pattern indicates a common structural strategy among various AAA+ ATPases, whereby hexameric arrangements and helix formations contribute to their functional mechanisms (Fig. 5).

### 3.4 MSMEG_0748 retains catalytic activity with a conserved Walker B motif

**Fig. 6:**
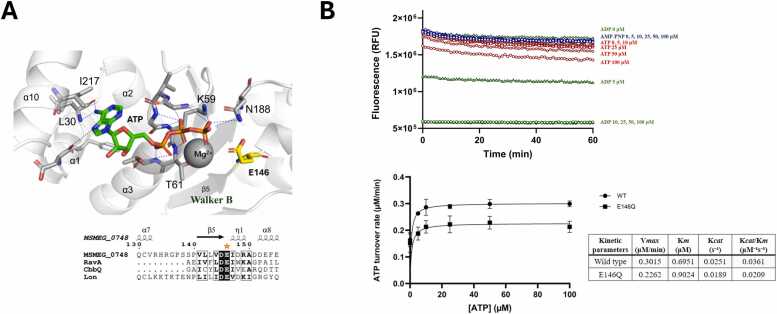
**Structural and functional analysis of the Walker B motif mutant (E146Q).** (A) Predicted structure of the ATP-binding pocket highlighting the Walker B motif. The ATP molecule (green) and coordinated Mg²⁺ ion (grey sphere) is shown, with interacting residues indicated (L30, K59, T61, E146, N188, and I217). The conserved glutamate residue (E146), located within the Walker B motif, is positioned to coordinate the Mg²⁺ ion and participate in ATP hydrolysis. A sequence alignment of MSMEG_0748 with homologous MoxR ATPases shows the conserved Walker B motif. (B) (Top Panel) The ATP/NADH coupled ATPase assay was used to measure ATP hydrolysis. MSMEG_0748 WT activity is strictly dependent on ATP, showing negligible rates with AMP-PNP and ADP. (Bottom Panel) The Michaelis-Menten plot displays ATP turnover rates for WT (black circles) and E146Q (open squares). The inset table summarizes the kinetic parameters. Error bars represent standard deviations from three independent experiments.

ATP hydrolysis is a fundamental function of AAA+ ATPases, and the catalytic glutamate residue within the Walker B motif plays a pivotal role in this process [24], [25]. Multiple sequence alignment with representative MoxR family ATPases confirmed that MSMEG_0748conserves this glutamate, corresponding to Glu146. This residue is located within the Walker B motif and is conserved across other MoxR-type ATPases. In the AlphaFold-predicted structure, the ATP molecule (green) is bound between the Walker A (red) and Walker B (orange) motifs, coordinated by a Mg²⁺ ion (gray sphere). The side chain of Glu146 (highlighted in yellow) resides in close proximity to the γ-phosphate of ATP, a spatial configuration that supports its role in nucleophilic attack during hydrolysis (Fig. 6A).

To functionally validate the importance of this residue and confirm the specificity of the reaction, we performed an ATP/NADH-coupled ATPase assay comparing wild-type MSMEG_0748 with the E146Q mutant, in which the glutamate is substituted with glutamine. The wild-type protein exhibited robust ATPase activity, which was strictly dependent on the presence of ATP. Critically, the non-hydrolyzable ATP analog AMP-PNP and the reaction product ADP resulted in negligible activity. Specifically, the rate of NADH oxidation, which is coupled to ATP hydrolysis in this assay, remained near zero across all tested ADP concentrations (10–100 μM). This observation confirms that MSMEG_0748 functions specifically as an ATPase and is unable to utilize the reaction product ADP as a substrate, consistent with canonical AAA+ ATPases (Fig. 6B, upper panel).

Furthermore, Michaelis-Menten kinetics were performed using varying ATP concentrations. The wild-type MSMEG_0748 exhibited an ATPase activity characterized by a Vmax of 0.3015 μM/min, a Km of 0.6951 μM, and a catalytic turnover rate (Kcat) of 0.0251 S^−1^. The E146Q mutant displayed significantly impaired activity. Its Vmax was reduced to 0.2262 μM/min (a ∼25 % decrease), and its Km was slightly increased to 0.9024 μM. More critically, the Kcat of the E146Q mutant decreased to 0.0189 S^−1^. Consequently, the overall catalytic efficiency (Kcat/Km) of the E146Q mutant (0.0209 S^−1^μM^−1^) was ∼42 % lower than that of the wild-type (0.0361 S^−1^μM^−1^). These pronounced kinetic changes demonstrate that MSMEG_0748 possesses intrinsic ATP hydrolysis activity that is critically dependent on the conserved Glu146 within the Walker B motif, as expected for canonical AAA+ ATPases (Fig. 6B, lower panel).

### 3.5 MSMEG_0748 interacts with MSMEG_0751 to facilitate CO-DH maturation

Our previous structural and biochemical analyses established MSMEG_0748 as a canonical MoxR-type AAA+ ATPase with intrinsic ATP hydrolysis activity. Typically, MoxR proteins require a VWA domain-containing partner to execute their functions in protein complex assembly or metalloenzyme maturation.

**Fig. 7:**
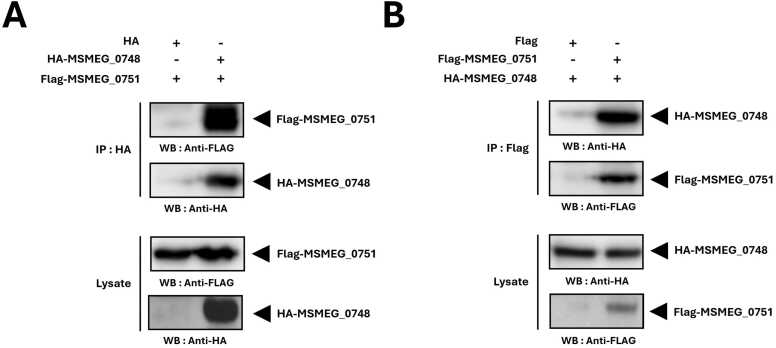
**MSMEG_0748 interacts with MSMEG_0751.** (A) Co-IP of HA-tagged MSMEG_0748, followed by Western blotting with anti-FLAG antibody, showing co-precipitation of Flag-MSMEG_0751. (B) Reciprocal Co-IP of Flag-tagged MSMEG_0751, followed by Western blotting with anti-HA antibody, showing co-precipitation of HA-MSMEG_0748. Lysate panels show total protein levels. These reciprocal Co-IP results confirm a direct interaction between MSMEG_0748 and MSMEG_0751, forming a stable MoxR-VWA complex.

To identify the physical partner of MSMEG_0748, we investigated MSMEG_0751, the VWA domain-containing protein encoded immediately adjacent to MSMEG_0748 in the same gene cluster of *M. smegmatis*. Co-IP experiments were conducted to assess a direct physical interaction between these components. Upon immunoprecipitation of HA-tagged MSMEG_0748 (IP: HA), Flag-tagged MSMEG_0751 was successfully detected in the eluate by Westen blotting (WB: Anti-FLAG) (Fig. 7). Conversely, immunoprecipitation of Flag-MSMEG_0751 (IP: Flag) also effectively pulled down HA-MSMEG_0748 (WB: Anti-HA). These reciprocal Co-IP results confirm that MSMEG_0748 physically interacts with MSMEG_0751.

To contextualize this interaction, we examined the genomic context surrounding the *msmeg_0748*-*msmeg_0751* gene pair. The gene pair encoding MSMEG_0748 and MSMEG_0751 is situated immediately downstream of the carbon monoxide dehydrogenase (CO-DH) gene cluster (*rv0377–rv0368c*) in *M. tuberculosis* and similarly co-localized in *M. smegmatis* (Fig. 1). Such co-localization of a putative metalloenzyme (CO-DH) and its associated AAA+ ATPase/VWA chaperone system is a recurring theme in prokaryotic regulatory modules. For example, in *Paracoccus denitrificans*, the MoxR protein NorQ, together with the VWA protein NorD, facilitates the insertion of an iron-sulfur cluster into cytochrome NO reductase [26]. In *E. coli*, RavA and ViaA are involved in the activation of fumarate reductase [27], while MoxR–MoxC/L pairs support calcium insertion in methanol dehydrogenase [28]. A distinct mechanism is observed in the Rubisco activation system, where CbbQ (MoxR) and CbbO (VWA) are thought to promote structural remodeling rather than metal insertion [29], [30]. These established systems suggest that MoxR–VWA complexes employ diverse mechanisms tailored to the target enzyme, strongly implying a functional link between the MSMEG_0748–MSMEG_0751 complex and CO-DH maturation, a link that is further supported by the high conservation and co-localization of the *msmge_0748–msmge_0751* gene pair across the *Mycobacterium* genus.

## 4 Conclusion

In this study, we characterized MSMEG_0748, a putative MoxR ATPase encoded within proximity the CO-DH gene cluster in *Mycobacterium smegmatis* MC^2^ 155. Utilizing sequence alignment, SEC-MALS, AlphaFold-based structural modeling, and ATPase activity assays, we demonstrated that MSMEG_0748 exhibits conserved features characteristic of the MoxR family ATPase, including hexameric assembly, the Walker B motif, and ATP hydrolysis activity. Structural analysis further revealed the presence of a characteristic Helix 2 Insert (H2I) extending into the central pore, consistent with previously reported MoxR architectures. Functional validation through site-directed mutagenesis of Glu146 confirmed its important role in ATP hydrolysis. Furthermore, Co-IP experiments demonstrated a physical interaction between MSMEG_0748 and its neighboring VWA domain-containing protein, supporting the hypothesis that these proteins function cooperatively in the maturation process of CO-DH. Collectively, our findings provide structural and functional insights into a previously uncharacterized mycobacterial MoxR ATPase, establishing a foundation for future investigations into alternative mechanisms of metalloenzyme activation within *Mycobacterium* species.

## CRediT authorship contribution statement

**So Yeon Lee:** Writing – original draft, Methodology, Investigation. **Hyo Guk Kim:** Methodology. **cho hyo je:** Writing – review & editing, Writing – original draft, Visualization, Validation, Supervision, Software, Resources, Project administration, Investigation, Funding acquisition, Data curation, Conceptualization. **Ju Eun Lee:** Writing – review & editing, Writing – original draft, Validation, Software, Methodology, Data curation. **Hyun-A Seong:** Validation, Methodology, Investigation, Data curation. **Sang-Min Jang:** Validation, Supervision, Data curation, Conceptualization. **Minkyung Cho:** Methodology, Data curation.

## Declaration of Competing Interest

The authors declare that they have no known competing financial interests or personal relationships that could have appeared to influence the work reported in this paper.

## References

[1] Kunau W.H., Beyer A., Franken T., Gotte K., Marzioch M., Saidowsky J., Skaletz-Rorowski A., Wiebel F.F. (1993). Two complementary approaches to study peroxisome biogenesis in Saccharomyces cerevisiae: forward and reversed genetics. Biochimie.

[2] Iyer L.M., Leipe D.D., Koonin E.V., Aravind L. (2004). Evolutionary history and higher order classification of AAA+ ATPases. J Struct Biol.

[3] Walker J.E., Saraste M., Runswick M.J., Gay N.J. (1982). Distantly related sequences in the alpha- and beta-subunits of ATP synthase, myosin, kinases and other ATP-requiring enzymes and a common nucleotide binding fold. EMBO J.

[4] Seraphim T.V., Houry W.A. (2020). AAA+ proteins. Curr Biol.

[5] Leipe D.D., Koonin E.V., Aravind L. (2003). Evolution and classification of P-loop kinases and related proteins. J Mol Biol.

[6] Ogura T., Wilkinson A.J. (2001). AAA+ superfamily ATPases: common structure--diverse function. Genes Cells.

[7] Ogura T., Whiteheart S.W., Wilkinson A.J. (2004). Conserved arginine residues implicated in ATP hydrolysis, nucleotide-sensing, and inter-subunit interactions in AAA and AAA+ ATPases. J Struct Biol.

[8] Wong K.S., Houry W.A. (2012). Novel structural and functional insights into the MoxR family of AAA+ ATPases. J Struct Biol.

[9] Neuwald A.F., Aravind L., Spouge J.L., Koonin E.V. (1999). AAA+: A class of chaperone-like ATPases associated with the assembly, operation, and disassembly of protein complexes. Genome Res.

[10] Bhandari V., Van Ommen D.A.J., Wong K.S., Houry W.A. (2022). Analysis of the evolution of the MoxR ATPases. J Phys Chem A.

[11] Bhandari V., Reichheld S.E., Houliston S., Lemak A., Arrowsmith C.H., Sharpe S., Houry W.A. (2023). The RavA-ViaA chaperone complex modulates bacterial persistence through its association with the fumarate reductase enzyme. J Biol Chem.

[12] Kahle M., Ter Beek J., Hosler J.P., Adelroth P. (2018). The insertion of the non-heme Fe(B) cofactor into nitric oxide reductase from P. denitrificans depends on NorQ and NorD accessory proteins. Biochim Biophys Acta Bioenerg.

[13] Tsai Y.C., Ye F., Liew L., Liu D., Bhushan S., Gao Y.G., Mueller-Cajar O. (2020). Insights into the mechanism and regulation of the CbbQO-type Rubisco activase, a MoxR AAA+ ATPase. Proc Natl Acad Sci USA.

[14] Kiazyk S., Ball T.B. (2017). Latent tuberculosis infection: an overview. Can Commun Dis Rep.

[15] Zacharia V.M., Shiloh M.U. (2012). Effect of carbon monoxide on Mycobacterium tuberculosis pathogenesis. Med Gas Res.

[16] Park S.W., Hwang E.H., Park H., Kim J.A., Heo J., Lee K.H., Song T., Kim E., Ro Y.T., Kim S.W., Kim Y.M. (2003). Growth of mycobacteria on carbon monoxide and methanol. J Bacteriol.

[17] Kim Y.M., Hegeman G.D. (1983). Oxidation of carbon monoxide by bacteria. Int Rev Cytol.

[18] Kim Y.M., Park S.W. (2012). Microbiology and genetics of CO utilization in mycobacteria. Antonie Van Leeuwenhoek.

[19] Dobbek H., Gremer L., Meyer O., Huber R. (1999). Crystal structure and mechanism of CO dehydrogenase, a molybdo iron-sulfur flavoprotein containing S-selanylcysteine. Proc Natl Acad Sci USA.

[20] Abramson J., Adler J., Dunger J., Evans R., Green T., Pritzel A., Ronneberger O., Willmore L., Ballard A.J., Bambrick J., Bodenstein S.W., Evans D.A., Hung C.C., O'Neill M., Reiman D., Tunyasuvunakool K., Wu Z., Zemgulyte A., Arvaniti E., Beattie C., Bertolli O., Bridgland A., Cherepanov A., Congreve M., Cowen-Rivers A.I., Cowie A., Figurnov M., Fuchs F.B., Gladman H., Jain R., Khan Y.A., Low C.M.R., Perlin K., Potapenko A., Savy P., Singh S., Stecula A., Thillaisundaram A., Tong C., Yakneen S., Zhong E.D., Zielinski M., Zidek A., Bapst V., Kohli P., Jaderberg M., Hassabis D., Jumper J.M. (2024). Accurate structure prediction of biomolecular interactions with AlphaFold 3. Nature.

[21] Elfmann C., Stülke J. (2023). PAE viewer: a webserver for the interactive visualization of the predicted aligned error for multimer structure predictions and crosslinks. Nucleic Acids Res..

[22] El Bakkouri M., Gutsche I., Kanjee U., Zhao B., Yu M., Goret G., Schoehn G., Burmeister W.P., Houry W.A. (2010). Structure of RavA MoxR AAA+ protein reveals the design principles of a molecular cage modulating the inducible lysine decarboxylase activity. Proc Natl Acad Sci USA.

[23] Hanzelmann P., Buchberger A., Schindelin H. (2011). Hierarchical binding of cofactors to the AAA ATPase p97. Structure.

[24] Jessop M., Felix J., Gutsche I. (2021). AAA+ ATPases: structural insertions under the magnifying glass. Curr Opin Struct Biol.

[25] Miller J.M., Enemark E.J. (2016). Fundamental Characteristics of AAA+ Protein Family Structure and Function. Archaea.

[26] Kahle M., Appelgren S., Elofsson A., Carroni M., Ädelroth P. (2023). Insights into the structure-function relationship of the NorQ/NorD chaperones from reveal shared principles of interacting MoxR AAA plus /VWA domain proteins. Bmc Biol.

[27] Wong K.S., Bhandari V., Janga S.C., Houry W.A. (2017). The RavA-ViaA chaperone-like system interacts with and modulates the activity of the fumarate reductase respiratory complex. J Mol Biol.

[28] Wong K.S., Snider J.D., Graham C., Greenblatt J.F., Emili A., Babu M., Houry W.A. (2014). The MoxR ATPase RavA and its cofactor ViaA interact with the NADH:ubiquinone oxidoreductase I in Escherichia coli. PLoS One.

[29] Tsai Y.C., Lapina M.C., Bhushan S., Mueller-Cajar O. (2015). Identification and characterization of multiple rubisco activases in chemoautotrophic bacteria. Nat Commun.

[30] Sutter M., Roberts E.W., Gonzalez R.C., Bates C., Dawoud S., Landry K., Cannon G.C., Heinhorst S., Kerfeld C.A. (2015). Structural characterization of a newly identified component of alpha-carboxysomes: the AAA+ domain protein CsoCbbQ. Sci Rep.

